# Lipopolysaccharide mediates immuno-pathological alterations in young chicken liver through TLR4 signaling

**DOI:** 10.1186/s12865-017-0199-7

**Published:** 2017-02-27

**Authors:** Xi-Yao Huang, Abdur Rahman Ansari, Hai-Bo Huang, Xing Zhao, Ning-Ya Li, Zhi-Jian Sun, Ke-Mei Peng, Juming Zhong, Hua-Zhen Liu

**Affiliations:** 1 Department of Basic Veterinary Medicine, College of Animal Science and Veterinary Medicine, Huazhong Agricultural University, Wuhan, Hubei 430070 China; 2 Section of Anatomy and Histology, Department of Basic Sciences, College of Veterinary and Animal Sciences (CVAS) Jhang, University of Veterinary and Animal Sciences (UVAS), Lahore, Pakistan; 3 Department of Anatomy, Physiology and Pharmacology, College of Veterinary Medicine, Auburn University, Auburn, USA

**Keywords:** Lipopolysaccharide, Chicken, Liver, Acute injury, Toll-like receptor 4

## Abstract

**Background:**

Lipopolysaccharide (LPS) induces acute liver injury and the complex mechanisms include the activation of toll like receptor 4 (TLR4) signaling pathway in many species. However, immuno-pathological changes during TLR4 signaling under LPS stress in acute liver injury is poorly understood in avian species. The present investigation was therefore carried out to evaluate these alterations in TLR4 signaling pathway during acute liver injury in young chickens.

**Results:**

After intraperitoneal injection of LPS or saline, liver samples were harvested at 0, 2, 6, 12, 24, 36, 72 and 120 h (*n* = 6 at each time point) and the microstructures were analyzed by hematoxylin and eosin (H&E) staining. Alanine aminotransferase (ALT) and caspase-3 enzyme activity was assessed by enzyme-linked immunosorbent assay (ELISA). Proliferative cell nuclear antigen (PCNA), single stranded DNA (ssDNA) and TLR4 protein expressions were determined by immunohistochemistry. Gene expressions of PCNA, caspase-3, caspase-8, TLR4 and its downstream molecules were analyzed by quantitative polymerase chain reaction (qPCR). LPS injection induced significantly higher ALT activity, severe fatty degeneration, necrotic symptoms, ballooning degeneration, congestion, enhanced inflammatory cell infiltration in liver sinusoids, decreased proliferation, increased apoptosis and significant up-regulation in TLR4 and its downstream molecules (MyD88, NF-κB, TNF-α, IL-1β and TGF-β) expression at different time points.

**Conclusions:**

This study indicated that TLR4 signaling and its downstream molecules along with certain cytokines play a key role in acute liver injury in young chickens. Hence, our findings provided novel information about the histopathological, proliferative and apoptotic alterations along with changes in ALT and caspase-3 activities associated with acute liver injury induced by *Salmonella* LPS in avian species.

**Electronic supplementary material:**

The online version of this article (doi:10.1186/s12865-017-0199-7) contains supplementary material, which is available to authorized users.

## Background

The liver is regarded as both metabolic as well as immunological lymphoid organ [1, 2]. It harbors many kinds of resident immune cells and has capability for the production of immune related defense mediators as well as regulatory molecules [3]. It is responsible for the synthesis of cytokines, chemokines, complement components and acute phase proteins that play essential role in innate immunity [3]. It is located at hemodynamic converging place in the body and conjoins the arterial system with portal venous system causing mixing of oxygenated blood with portal venous blood. The liver sinusoids have several components of nutrients, lymphocytes and myeloid cells together with many kinds of antigens and other microbial products as derived from intestinal bacteria [4, 5]. The liver is also under constant exposure of environmental toxins, food antigens and bacterial components [6]. Lipopolysaccharride (LPS) or endotoxin is a major component of cell wall in Gram negative bacteria. Under normal physiological conditions, LPS is not detectable in systemic blood circulation. However its detectable amount (about 1.0 ng/ml) is usually present in portal venous circulation [5]. LPS stimulation has been widely used in several experimental models [7–9] for the understanding of mechanisms involved in endotoxin-mediated acute liver tissue damage [7]. However, LPS-induced immuno-pathological and micro-morphological alterations in chicken liver are poorly understood yet.

Toll like receptors (TLRs) are considered as evolutionary conserved pattern recognition receptors (PPRs) that act as critical mediators of host response to many pathogenic organisms [10, 11]. PPRs identify the pathogen associated molecular patterns (PAMPs) and the appropriate localization of TLRs in cells is considered to be important for the accessibility of ligand and the understanding of downstream signal transduction molecules [12]. Until now 13 functional TLRs have been reported in mouse [13] and as many as 10 in both human [12] and chicken [14]. Out of these, TLR4 plays an important role after LPS stimulation and induces host defense mechanism that leads to the activation of intracellular signaling pathways and production of co-stimulatory molecules and cytokines [9, 15]. TLR4 expression has been reported in both parenchymal and non-parenchymal liver cells in response to injury [16]. Parenchymal cells of liver undergo apoptotic changes during liver injury [17]. Deregulation of transforming growth factor β (TGF-β) is also associated with liver cancer and fibrotic liver disease. Activation of TGF-β signaling pathway leads to immune suppression, arrest of cell cycle at G1/S phase and induction of apoptosis in mouse model [18]. But the information about the changed expression of these cytokines in chicken liver under endotoxin stress is still scarce. Moreover, proliferative and apoptotic changes during TLR4 signaling need further characterization at tissue level in LPS-induced chicken liver. Therefore, the current study was designed for the better understanding of micro-morphological changes and molecular events involved in TLR4 mediated hepatic injury following intrapertoneal LPS stimulation in time series manner in young chickens.

## Methods

Healthy one-day-old commercial Cobb strain (genetically Cobb 500) broiler chicks were purchased from Zhengda chicken breeding company (Wuhan, China) and chicks with uniform body weight were selected and provided with commercial chick-starter feed and water *ad libitum* along with supplementary heating without any vaccinations [19]. All the birds were intraperitoneally (i.p.) injected at the same peritoneal location by lifting the skin over mid-abdominal line, immediately anterior to the pubic bones with LPS derived from *Salmonella* enterica serovar Typhimurium (STm) (L7261; Sigma-Aldrich, St. Louis, MO, USA) at 50 mg/kg of body weight in 0.5 mL avian saline solution (0.75% NaCl) [19]. Birds in the control group were exposed to mock infection with 0.5 mL avian saline solution only.

The chickens (*n =* 6 at each time point) were euthanized by CO_2_ inhalation and sacrificed by dissecting the abdominal cavity at 0, 2, 6, 12, 24, 36, 72 and 120 h. After dissection, liver samples were immediately harvested from the birds for morphological and molecular studies. A portion of liver samples were fixed in 4% paraformaldehyde solution in PBS, dehydrated and then embedded in paraffin wax for morphological analysis. After that, 4-μm tissue sections were cut using a Leica microtome (Nussloch Gmbh, Germany) and mounted on polylysine-coated slides (Boster Corporation, China). The rest of fresh liver samples were also frozen quickly in liquid nitrogen and then stored at −70 °C for qPCR and ELISA analysis.

H&E staining was performed by routinely used protocol. Stained tissue sections were examined by light microscopy (Olympus BX51, Tokyo, Japan) with a digital camera (DP72; Olympus).

The tissue sections were immunostained by following the same steps as described previously [19, 20]. In brief, serial liver tissue sections were deparaffinized twice in xylene and rehydrated in a graded series of ethanol. Heat antigen retrieval was accomplished using a microwave oven (MYA-2270 M, Haier, Qindao, China) and tissue sections were microwaved in citrate acid buffer solution (pH 6.0) for 20 min (5 min at high level i.e., 700 W and 15 min at low level i.e., 116 W). Following heat-induced antigen retrieval, tissue section were allowed to cool down at room temperature for 2–3 h. Endogenous peroxidase activity was quenched by treating tissue sections with 3% H_2_O_2_ for 10 min at room temperature. To block non-specific antibody binding, the tissue sections were then incubated with 5% bovine serum albumin (BSA) at 37 °C for half an hour. Liver tissue sections were then incubated with primary antibodies using rabbit anti-TLR4 antibody (1:100) and PCNA (1:200) (Santa Cruz Biotechnology, Inc., Santa Cruz, CA, USA). Subsequently, tissue sections were incubated at 37 °C with suitable horseradish peroxidase (HRP)-conjugated secondary antibodies (Boster, Wuhan, China) for 30 min. In situ detection of cell apoptosis was accomplished by using a mouse IgM anti-ssDNA monoclonal antibody (1:30; EMD Millipore, Billerica, USA), following same steps as described above with the exception of treatment of tissue sections with 0.1 mg/ml saponin and 20 μg/ml proteinase K in PBS for 20 min at 37 °C, incubation in 50% (v/v) formamide in distilled water for 20 min at 56 °C. These sections were then cooled in cold PBS for 5 min, instead of heat induced antigen retrieve in a micro oven, and employed anti-mouse IgM SABC kit (Boster, Wuhan, China) instead of other secondary antibodies kit. Immunostaining for all the tissue sections was accomplished using chromogenic marker, diaminobenzidine (DAB) (Boster, Wuhan, China) and counterstaining was performed using hematoxylin. Finally, sections were washed, dried, dehydrated, cleared, and mounted with a coverslip. In the current study, isotype serum of primary antibodies was used for both LPS stimulated and saline treated (negative control) groups.

Serial sections were examined under a light microscope (BH-2; Olympus, Japan) with a digital camera (DP72; Olympus), and the fields of vision were chosen according to different regions of the liver tissue in each section. The distribution and expression level of different proteins were measured in high-power fields selected at random. All of the images were taken using the same microscope and camera set. Image-Pro Plus (IPP) 6.0 software (Media Cybernetics, USA) was used to calculate the integral optical density (IOD) for positive staining (Additional file 1) and the graphs were prepared by Prism software version 5.0 (GraphPad Software, Inc., San Diego, USA).

The expression level of alanine aminotransferase (ALT) and caspase-3 activity of liver tissues were determined by following previously described modified ELISA method [21].

The tissue homogenate for ALT activity assay was prepared according to the manufacturer’s instructions. Briefly, the samples of liver from the ultra-low temperature freezer were weighed and homogenized (0.1 g of tissue in 0.90 ml of 4 °C pre-cooled physiological saline). The homogenate was centrifuged at 1000 × g for 10 min and then aliquots supernatants were stored at −70 °C. The expression level of alanine aminotransferase (ALT) of liver tissues was assessed using ALT assay kit (C009-2, Nanjing Institute of Jiancheng, China). Briefly, standards and supernatants obtained from the processed liver tissues were pipetted into the wells. The absorbencies were read at 492 nm wave length. For each set of reference standards, samples and control, the average absorbance values (A_492_) were calculated with the help of standard curve.

The cell lysate for casepase-3 activity assay was prepared according to the manufacturer’s instructions. Briefly, the 100 mg solid liver tissues were cut into small pieces and then 100 μl lysate pre-cooled working fluid was added in an ice bath and homogenized with a glass homogenizer. Centrifugation was performed at 12,000 × g for 10 min at 4 °C and then the supernatant (lysate containing protein) was transferred to a new tube and placed on ice until needed. The expression level of caspase-3 activity in liver tissues was determined using caspase-3 activity assay kit (G007, Nanjing Institute of Jiancheng, China). Briefly, cell lysate obtained from the processed liver tissues and standard solutions from the kit were pipetted into the wells according to the recommended experimental setting. After 4 h incubation at 37 °C, the color changes were obvious. The absorbencies were measured at 405 nm on microplate reader. The final caspase-3 activity levels were determined by comparing optical density (OD) values from apoptosis inducer and negative control wells.

Total RNA was extracted from liver tissues according to the manufacturer’s instructions. Then total RNA were treated with RNase-free DNase I (Fermentas, Opelstrasse, Germany) to remove contaminating genomic DNA. The first strand cDNA was synthesized using the RevertAid First Strand cDNA Synthesis Kit (Fermentas, Opelstrasse, Germany). The reaction mixture (10 μl) for qPCR contained of 5 μL SYBR Select Master Mix for CFX (Applied Biosystems), 0.2 μL of each forward and reverse primer and 1 μL of template cDNA. The qPCR reactions were performed on a Bio-Rad CFX Connect real-time PCR detection system (Bio-Rad, Hercules, CA, USA). The qPCR conditions were as follows: pre-denaturation at 95 °C for 5 min, followed by 40 cycles of denaturation at 95 °C for 30 s, annealing at 60 °C for 30 s, and elongation at 72 °C for 20 s. The primer sequences used in this experiments are listed in Table 1. All samples were run in triplicate and gene expression levels were quantified (Additional file 2) using the ΔΔCt method [22].

**Table 1:** Primers used for Real-time PCR

Gene	Primer sequences (5′to3′)	Accession no.
actin beta	f-TTGTTGACAATGGCTCCGGT r-TCTGGGCTTCATCACCAACG	NM_205518.1
TLR4	f-TGAAAGAGCTGGTGGAACCC r-CCAGGACCGAGCAATGTCAA	NM_001030693.1
MyD88	f-AGGATGGTGGTCGTCATTTC r-TTGGTGCAAGGATTGGTGTA	NM_001030962.2
NF-κB	f-CTACTGATTGCTGCTGGAGTTG r-CTGCTATGTGAAGAGGCGTTGT	M86930.1
TNF-α	f-CAGATGGGAAGGGAATGAAC r-CACACGACAGCCAAGTCAAC	AY765397.1
IL-1β	f-ACCTACAAGCTAAGTGGGCG r-ATACCTCCACCCCGACAAGG	NM_204524.1
TGF-β	f-ATGTGTTCCGCTTTAACGTGTC r-GCTGCTTTGCTATATGCTCATC	NM_205454.1
caspase-3	f- TCCACCGAGATACCGGACTG r- ACAAAACTGCTTCGCTTGCT	NM_204725.1
caspase-8	f- CGGATCAATCGAATAGACCTTC r- CGGCATTGTAGTTTCAGGACTT	NM_204592.2
PCNA	f- TCTGAGGGCTTCGACACCTA r- AACCTTTTCCTGATTTGGTGCTT	NM_204170.2

**Fig. 1:**
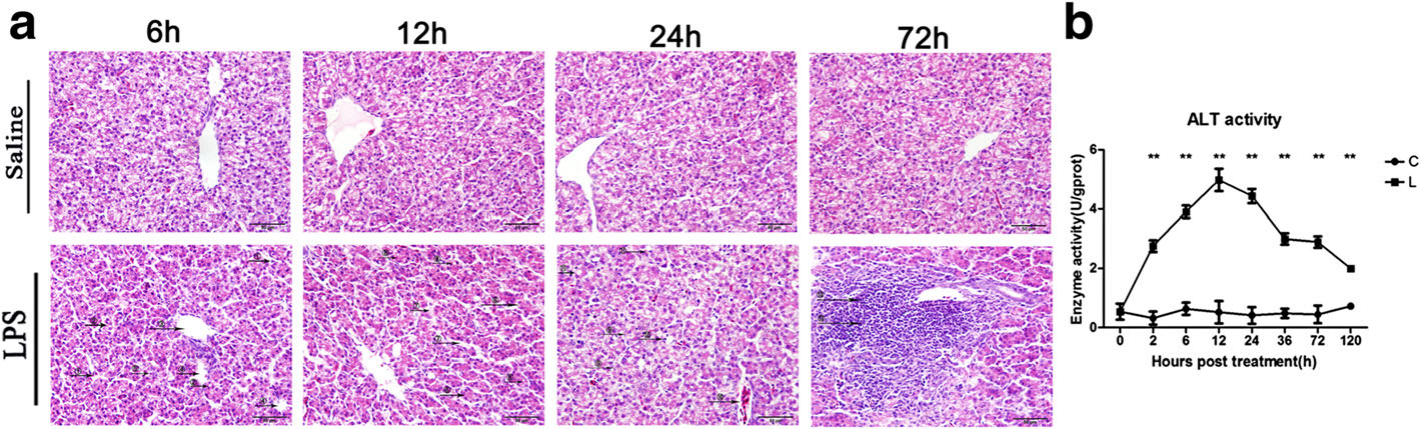
Effect of lipopolysaccharide on histomorphology and ALT activity and in chicken liver. Following intraperitoneal LPS treatment in chickens at different time points, H&E staining was performed on liver serial tissue sections. Stellate macrophages (Kupffer cells) in perisinusoidal areas ①, diffuse infiltration of fat vacuoles indicating fatty infiltration ②, dilated central vein ③ and sinusoidal capillaries ④, reduction in size of a few hepatocytes ⑤, dissociated liver cells from each other in hepatic cords ⑥, dilated hepatic sinusoids along with fibrocytes proliferation in perisinusoidal areas ⑦, intracytoplasmic infiltration of variable size and shape fat vacuoles ⑧, dilated hepatic sinusoids ⑨, infiltration of oval shaped nucleated RBCs ⑩, cytoplasmic fat vacuoles have pushed hepatocyte nuclei at periphery ⑪, reduction in size of a few hepatocytes ⑫ and intense inflammatory cells infiltration around the portal area ⑬ (**a**). After LPS stimulation, alanine aminotransferase (ALT) activity was measured from liver tissues at 0 h, 2 h, 6 h, 12 h, 24 h, 36 h, 72 h and 120 h by ELISA technique (**b**). The letter C represents saline (control) group and L represents LPS group. The numbers represent the hours after stimulation. **P < 0.01

**Fig. 2:**
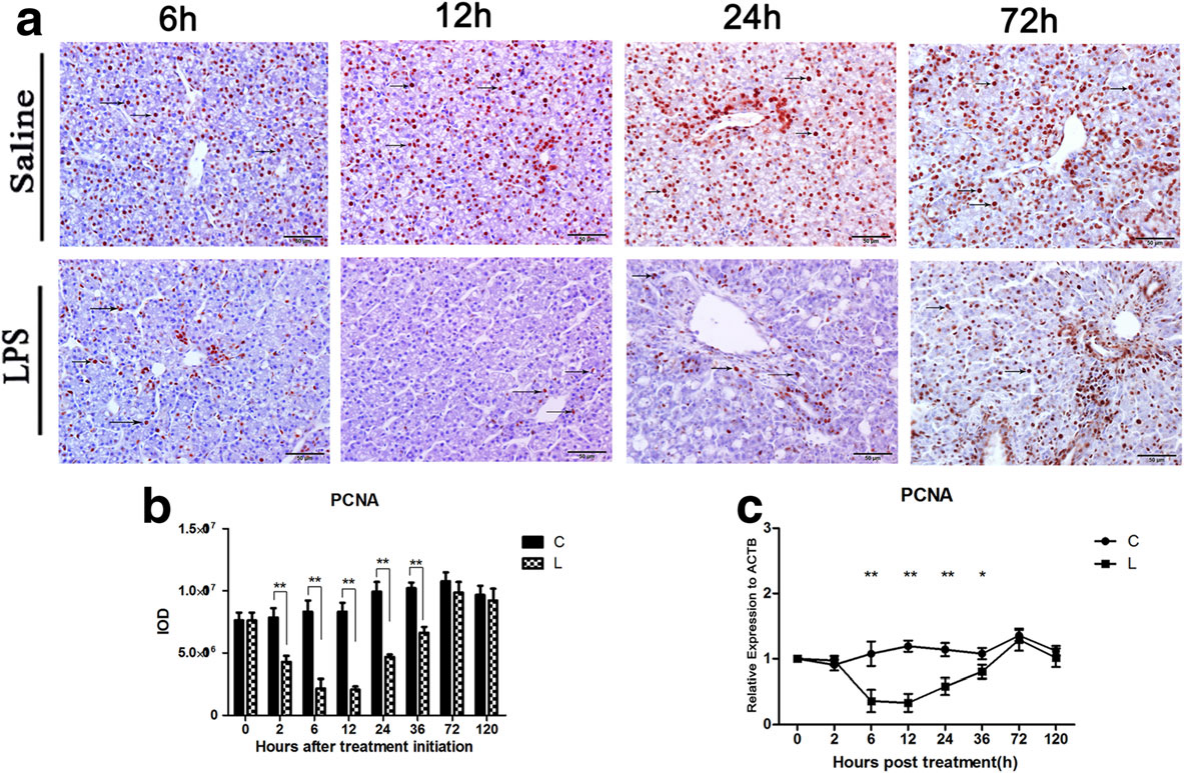
Effect of LPS stimulation on hepatic cell proliferation in chicken liver. After intraperitoneal LPS injection in chicks at different time points, PCNA protein expression was assessed in liver tissue by immunohistochemistry using anti-PCNA antibody, PCNA positive product was mainly distributed around the portal and biliary epithelial cells and more concentrated expression was present on epithelial cell near portal area in saline group at 6 h, 12 h, 24 h, and 72 h as compared to LPS group (**a**). Quantification of PCNA expression from liver tissue images was accomplished by image-pro plus (IPP) computer software where IOD represents integrated optical density (**b**). The analysis of PCNA gene expression was performed by real-time quantitative RT-PCR and normalized by the expression of actin beta (ACTB) (**c**). The letter C represents saline (control) group and L represents LPS group. The numbers represent the hours after stimulation. *P < 0.05, **P < 0.01

**Fig. 3:**
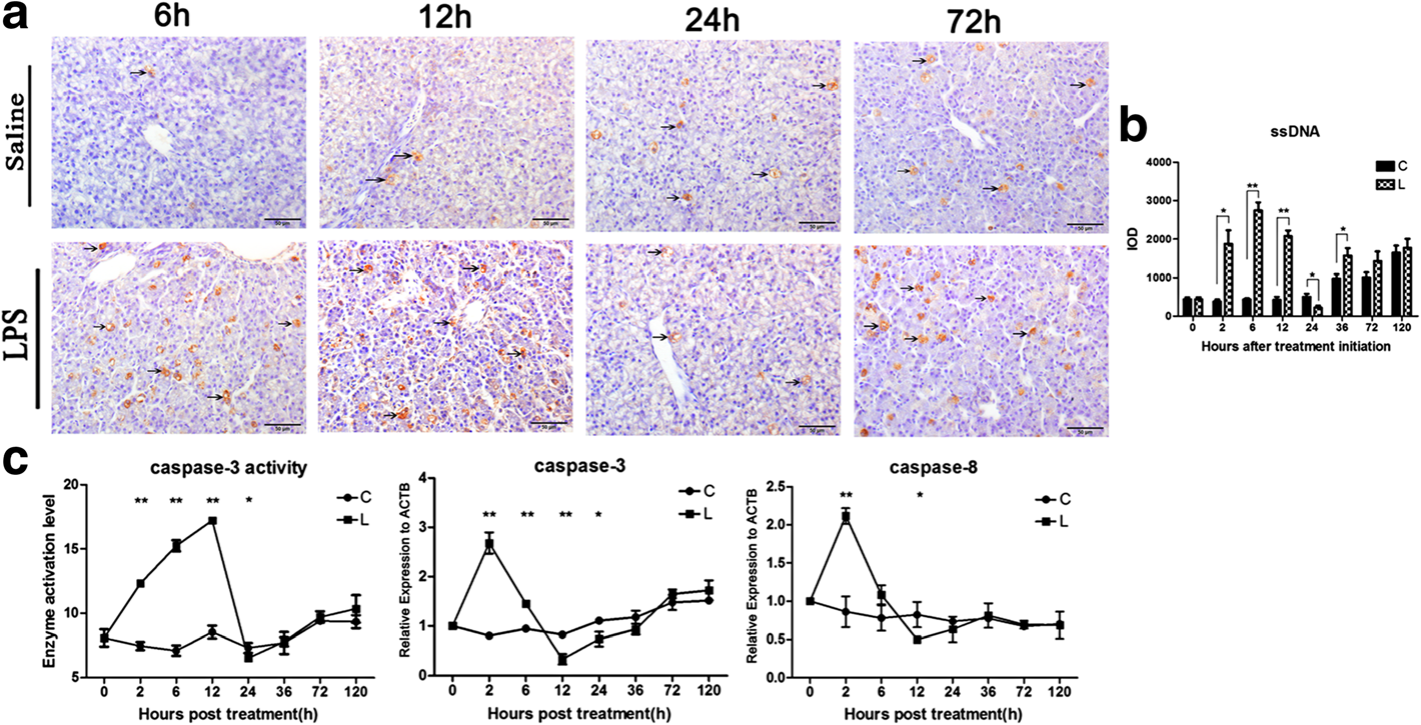
Effect of LPS stimulation on hepatocyte apoptosis in chicken liver. Following intraperitoneal LPS injection in chicks at different time points, single stranded DNA (ssDNA) protein expression was assessed in liver tissues by immunohistochemistry using anti-ssDNA antibody, ssDNA positive product was extensively distributed in biliary epithelial cells and hepatic sinosoidal endothelial cells in LPS group at 6 h, 12 h, 24 h, and 72 h as compared to PBS (saline) group (**a**). Quantification of ssDNA expression from liver tissue images was accomplished by image-pro plus (IPP) computer software where IOD represents integrated optical density (**b**). The activity of caspase-3 enzyme was measured by ELIZA technique and the expressions of caspase-3 and caspase-8 genes were also determined by quantitative RT-PCR and normalized by the expression of actin beta (ACTB) (**c**). The letter C represents saline (control) group and L represents LPS group. The numbers represent the hours after stimulation. *P < 0.05, **P < 0.01

**Fig. 4:**
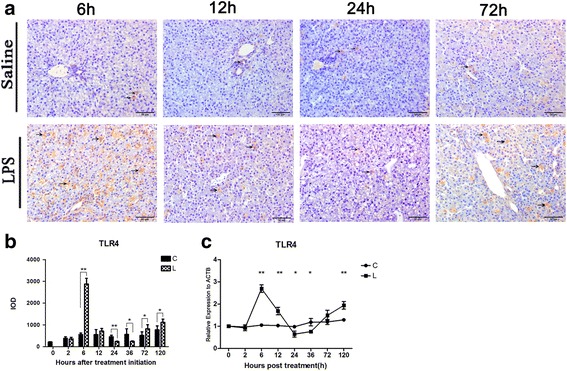
Effect of LPS stimulation on TLR4 expression in chicken liver. After intraperitoneal LPS injection in chicks at different time points, TLR4 protein expression was assessed in liver tissue by immunohistochemistry using anti-TLR4 antibody, TLR4 positive product was mainly distributed on hepatocytes. In LPS group, strong TLR4 expression was present at 6 h, 12 h, 24 h, and 72 h as compared to saline group (**a**). Quantification of TLR4 expression from liver tissue images was accomplished by image-pro plus (IPP) computer software where IOD represents integrated optical density (**b**). The analysis of TLR4 gene expression was performed by quantitative RT-PCR and normalized by the expression of actin beta (ACTB) (**c**). The letter C represents saline (control) group and L represents LPS group. The numbers represent the hours after stimulation. *P < 0.05, **P < 0.01

**Fig. 5:**
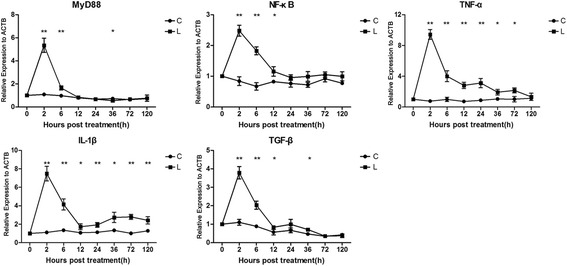
Effect of LPS stimulation on downstream molecules of TLR4 signaling and cytokines in chicken liver. Following intraperitoneal LPS stimulation in chicks at 0 h, 2 h, 6 h, 12 h, 24 h, 36 h, 72 h and 120 h, the expressions of MyD88, NF-κB, TNF-α, TGF-β and IL-1β genes were determined by real-time quantitative PCR (qRT-PCR) and normalized by the expression of actin beta (ACTB). The letter C represents saline (control) group and L represents LPS group. The numbers represent the hours after stimulation. *P < 0.05, **P < 0.01

Data were expressed as the mean ± standard deviation (SD) and the statistical analyses were performed using the GraphPad Prism version 5.0. The arithmetic mean was calculated and any significant differences between groups in the same tissue regions were analyzed using the independent-samples *t* test for group means (Fig. 2b, Fig. 3b and Fig. 4b). The statistical significance in the comparison of multiple sample sets versus control was performed with Bonferroni’s multiple comparisons test after one-way ANO VA test (Fig. 1b, Fig. 2c, Fig. 3c, Fig. 4c and Fig. 5). Differences were considered significant if *P* < 0.05. *P < 0.05, **P < 0.01 and ***P < 0.001.

## Results

### Acute liver injury after Salmonella lipopolysaccharide stimulation

In comparison to saline group, histopathology of liver showed prominent stellate macrophages (Kupffer cells) in peri-sinusoidal areas, diffuse infiltration of fat vacuoles indicating fatty infiltration, dilation of both central veins and sinusoidal capillaries and reduction in size of a few hepatocytes at 6 h post LPS stimulation. Liver cells were seen dissociated from each other in hepatic cords, hepatic sinusoids were dilated at many places along with fibrocyte proliferation in peri-sinusoidal areas and intracytoplasmic infiltration of variable size and shape fat vacuoles was seen at 12 h post LPS stimulation. Hepatic sinusoids were dilated in many areas with severe vascular congestion, cytoplasmic fat vacuoles have pushed hepatocyte nuclei at periphery at some places and reduction in size of a few hepatocytes was also seen at 24 h post LPS stimulation. Obvious pathological changes (inflammatory cell infiltration around the portal area) were present at 72 h post LPS stimulation (Fig. 1a; Additional file 3). Following LPS treatment, ALT activity was significantly higher than that of control group. It reached the peak at 12 h after LPS stimulation, and then gradually returned to normal level (Fig. 1b).

### PCNA expression was remarkably decreased after LPS stimulation in chicken liver

In the control (saline) group, the PCNA positive products showed brownish shades under microscope and mainly distributed around the portal and biliary epithelial cells and more concentrated expression was present on epithelial cell near portal area (Fig. 2a). PCNA expression was remarkably decreased after LPS stimulation (*P* < 0.01) at 6 h, 12 h, 24 h, and 36 h as compared to saline group (Fig. 2b). Consistent with the results of PCNA by immunohistochemistry, mRNA expression of PCNA following LPS stimulation exhibited first decrease and then slightly returned towards the normal level and showed significant difference at 6 h, 12 h, 24 h (*P* < 0.05) and 36 h (*P* < 0.01) as compared to control group (Fig. 2c).

### Effect of LPS stimulation on hepatocyte apoptosis

The single stranded DNA (ssDNA) positive products showed brownish shades under microscope and mainly distributed in the hepatocytes, biliary epithelial cells and hepatic sinosoidal endothelial cells (Fig. 3a). IPP analysis indicated that ssDNA expression changed after LPS stimulation and showed significant up-regulation (*P* < 0.05 or *P* < 0.01) at 2 h, 6 h, 12 h and 36 h, while significant down regulation (*P* < 0.05) at 24 h as compared to control group (Fig. 3b). The activity of caspase-3 as measured by ELISA, was considerably enhanced at 2 h, 6 h and 12 h (*P* < 0.01) in LPS stimulated group as compared to control group. The levels of mRNA expression of caspase-3 following LPS stimulation exhibited first increased, then decreased and again a little increased trends and showed significant increase at 2 h, 6 h (*P* < 0.01) and significant decrease at 12 h and 24 h (*P* < 0.05 or *P* < 0.01) as compared to control group. The statistical analysis of mRNA expression of caspase-8 following LPS stimulation exhibited similar events as of caspase-3 i.e., first increased, then slightly decreased and again a little increased trends and showed significant increase at 2 h (*P* < 0.01) and significant decrease at 12 h (*P* < 0.05) as compared to control group (Fig. 3c).

### Effect of LPS stimulation on TLR4 expression in chicken liver

TLR4 protein expression in chicken liver tissue sections was determined by immunoperoxidase–hematoxylin staining. In TLR4-positive hepatocytes, the cytoplasm and plasma membrane were stained light brown by DAB and nucleus was stained blue with hematoxylin. In control group, the weak TLR4 expression was only present in portal bile duct epithelial cells. After LPS stimulation TLR4 expression was more concentrated and presented in hepatocytes in the liver (Fig. 4a). IPP analysis showed that TLR4 expression was remarkably increased after LPS stimulation at 6 h, 72 h and 120 h (*P* < 0.05 or *P* < 0.01) ) while significantly decreased at 24 h and 36 h (*P* < 0.05) as compared to control group (Fig. 4b). The statistics of mRNA expression of TLR4 following LPS stimulation exhibited first increase, then decrease and again increase trends and showed significant increase at 6 h, 12 h and 120 h (*P* < 0.01) and significant decrease at 24 h and 36 h (*P* < 0.05) as compared to control group (Fig. 4c).

### Effect of LPS stimulation on downstream molecules of TLR4 signaling pathway and cytokines in chicken liver

Following LPS stimulation in chickens, the statistics of mRNA expression of MyD88 exhibited first drastic increase, then considerable decrease and again slight increase trends and showed very significant increase at 2 h and 6 h (*P* < 0.01) and significant decrease at 36 h (*P* < 0.05) while NF-κB demonstrated increasing trends at all the time points and showed significant difference at 2 h, 6 h and 12 h (*P* < 0.05 or *P* < 0.01) as compared to control group. The gene expressions of cytokines (TNF-α, TGF-β and IL-1β) exhibited increasing trends at all the time points after LPS stimulation illustrating significant difference (*P* < 0.05 or *P* < 0.01) at several time points as compared to saline group (Fig. 5).

## Discussion

Alanine aminotransferase (ALT) is an important liver enzyme and exists in cytosol of hepatocytes. ALT activity has been reported about 3000 times in liver tissues than that in serum. Increased level of ALT is present during acute hepatocellular injury, therefore the direct measurement of ALT activity is more efficient and accurate for the damaged liver tissue [23]. Bacterial LPS is well known and critical cofactor that is usually implicated in liver injury [24]. In previous studies, LPS stimulation was found to be linked with considerable increase in serum ALT release in both mice [25] and chicken [26]. In the current investigation, we found significantly higher ALT release in liver tissue after intraperitoneal LPS stimulation that attained its peak at 12 h of treatment in young chicken as compared to control group. All these facts indicated that LPS could disrupt liver function particularly at early stages of pathological stimulation.

LPS administration has been found to disrupt liver architecture and leads to significant alteration in histological organization along with fatty degenerations and irregular and loose arrangement of hepatic cells in mice [25]. In the present study, liver showed fatty degeneration, necrotic symptoms, ballooning degeneration, congestion and enhanced inflammatory cell infiltration at different time points of LPS treatment as compared to saline injected control group in young chickens. In a previous study, LPS treatment exhibited considerable morphological changes such as necrosis, lymphocytic infiltration, Kupffer cell hyperplasia and portal triaditis in murine experimental models [27]. Hence, it seems that LPS stimulation may cause similar histo-pathological alteration in both murine and chicken liver. However underlying molecular mechanism needs further investigation.

In this study, both mRNA levels and cellular expression of proliferative cell nuclear antigen (PCNA) by immunohistochemistry were remarkably decreased at certain time points after LPS stimulation. In a prior report, LPS stimulation also showed decreased expression of PCNA in murine model with acute liver damage [28]. It is also reported that LPS treatment can trigger the activation of apoptosis related genes and the activated caspase-3 ultimately causes the cell apoptosis [29, 30]. Herein, we found significant up regulation in the expression of apoptosis related genes, caspase-3 and caspase-8 at different time points after LPS stimulation. Moreover, single stranded DNA (ss-DNA) protein expressions by immunohistochemistry were also decreased significantly after LPS treatment in the current investigation. Previously hepatocyte apoptosis has been observed after intravenous treatment of LPS in experimental shock models and the activated caspase-3 in liver tissue corresponds to apoptotic index in hepatocytes [31, 32]. Hence, it is concluded that decreased proliferation and increased apoptosis are associated with LPS induced acute liver injury in young chickens.

Complex mechanisms are involved in LPS induced acute liver damage [33]. High expression of TLR4 and down streaming molecules such as MyD88 play an essential role in progression of LPS induced acute liver injury and act as powerful mediator of inflammatory process and innate immune activation [34–36]. Herein, strong TLR4 expression was present on hepatocytes in liver and both protein and mRNA expressions levels of TLR4 were remarkably increased at certain time points after LPS stimulation. Previously, liver mRNA of chicken was sequenced for the determination of the entire chTLRs (chicken TLRs) sequences [8]. The expression of TLR4 has been reported in activated hepatic stellate cells (HSCs) as well as on parenchymal and non-parenchymal hepatic cells during acute liver damage [16]. In the current study, mRNA expressions of MyD88 and NF-κB are significantly increased at certain time points following LPS stimulation in chicken liver. During TLR4 signaling, both myeloid differentiation primary response gene 88 (MyD88)-dependent and MyD88-independent pathways are activated upon LPS stimulation in mammals and MyD88-dependent pathway leads to production of transcription factors such as nuclear factor kappaB (NF-kB) along with expressions of tumor necrosis factor (TNF) and interleukin (IL) while MyD88-independent pathway arbitrates the induction of type-I interferones and interferon-inducible genes [37–39]. In contrast, only MyD88-dependent signaling is involved in response to TLR4-MD2 complex activation under LPS stress in chicken [9]. Therefore, it is concluded that LPS/TLR4-MyD88-dependent signaling along with its downstream molecules is involved in acute liver injury in young chickens.

Determination of cytokine expressions during bacterial infection not only helps in the understanding of appropriate induction of immune response but also assists in the devising innovative therapeutic strategies [40]. In the present investigation, the statistical analysis of mRNA expression of different inflammatory cytokines such as TNF-α, IL-1β and TGF-β showed significant increase at different time points after LPS treatment in young chicken liver. In a previous study, several inflammatory cytokines such as interleukin-1 (IL-1), tumor necrosis factor-α (TNF-α) along with reactive oxygen intermediates are produced by liver in response to LPS exposure and play critical roles in its injury [24]. Hepatic injury in response to LPS exposure is also caused by TNF-α that is secreted from Kupffer cells [31]. Taken together, it is concluded that LPS/TLR4 signaling along with its downstream molecules and cytokines may play key role in acute liver injury in avian species.

## Conclusions

This study demonstrated that LPS is involved in acute liver injury and significantly altered the liver’s structure and function in young chickens. It was found that TLR4 signaling and its downstream molecules along with certain cytokines play a key role in hepatocyte apoptosis during acute liver injury in young chickens. Hence, our findings provided novel information about the histopathological, proliferative and apoptotic alterations along with changes in ALT and caspase-3 activities associated with acute liver injury induced by Salmonella LPS in avian species.

## Additional files

Additional file 1: Integral optical density (IOD) values for TLR4, anti-ssDNA & PCNA expression. (XLSX 13 kb)
Additional file 2: Relative values of genes for qPCR expression. (XLSX 20 kb)
Additional file 3: Images of Fig. 1a, at 6 h, 12 h, 24 h and 72 h post LPS stimulation. (PDF 1713 kb)

## Abbreviations

ALT: Alanine aminotransferase
BSA: Bovine serum albumin
CFU: Colony forming unit
DAB: 3,3′-diaminobenzidine
ELIZA: Enzyme-linked immunosorbent assay
G1: Growth 1 phase
H&E: Hematoxylin and Eosin
h: Hour/hours
HRH: Horseradish peroxidase
IHC: Immunohistochemistry
IL: Interleukin
IOD: Integrated optical density
IPP: Image-Pro-plus
LPS: Lipopolysaccharide
MyD88: Myeloid differentiation primary response gene 88
NF-kB: Nuclear factor-κB
PAMPs: Pathogen associated molecular patterns
PBS: Phosphate buffered saline
PCNA: Proliferating cell nuclear antigen
PRRs: Pattern recognition receptors
qRT-PCR: Quantitative real time polymerase chain reaction
S phase: DNA synthesis phase
SABC: StreptAvidin-Biotin Complex
SD: Standard deviation
ssDNA: Single stranded DNA
STm: Salmonella enterica serovar Typhimurium
TLRs: Toll like receptors
TNF-α: Tumour necrosis factor-alpha
