# Supplementary material for: Lipopolysaccharide mediates immuno-pathological alterations in young chicken liver through TLR4 signaling
Source: BMC Immunol. 2017 Feb 27;18:12. doi: 10.1186/s12865-017-0199-7 (PMC5327529; doi:10.1186/s12865-017-0199-7)
Supplement: Additional file 3: — Images of Fig. 1a, at 6 h, 12 h, 24 h and 72 h post LPS stimulation. (PDF 1713 kb) [file 12865_2017_199_MOESM3_ESM.pdf]

Additional file 1: Images of Fig. 1a at 6h, 12h, 24h and 72h post LPS stimulation

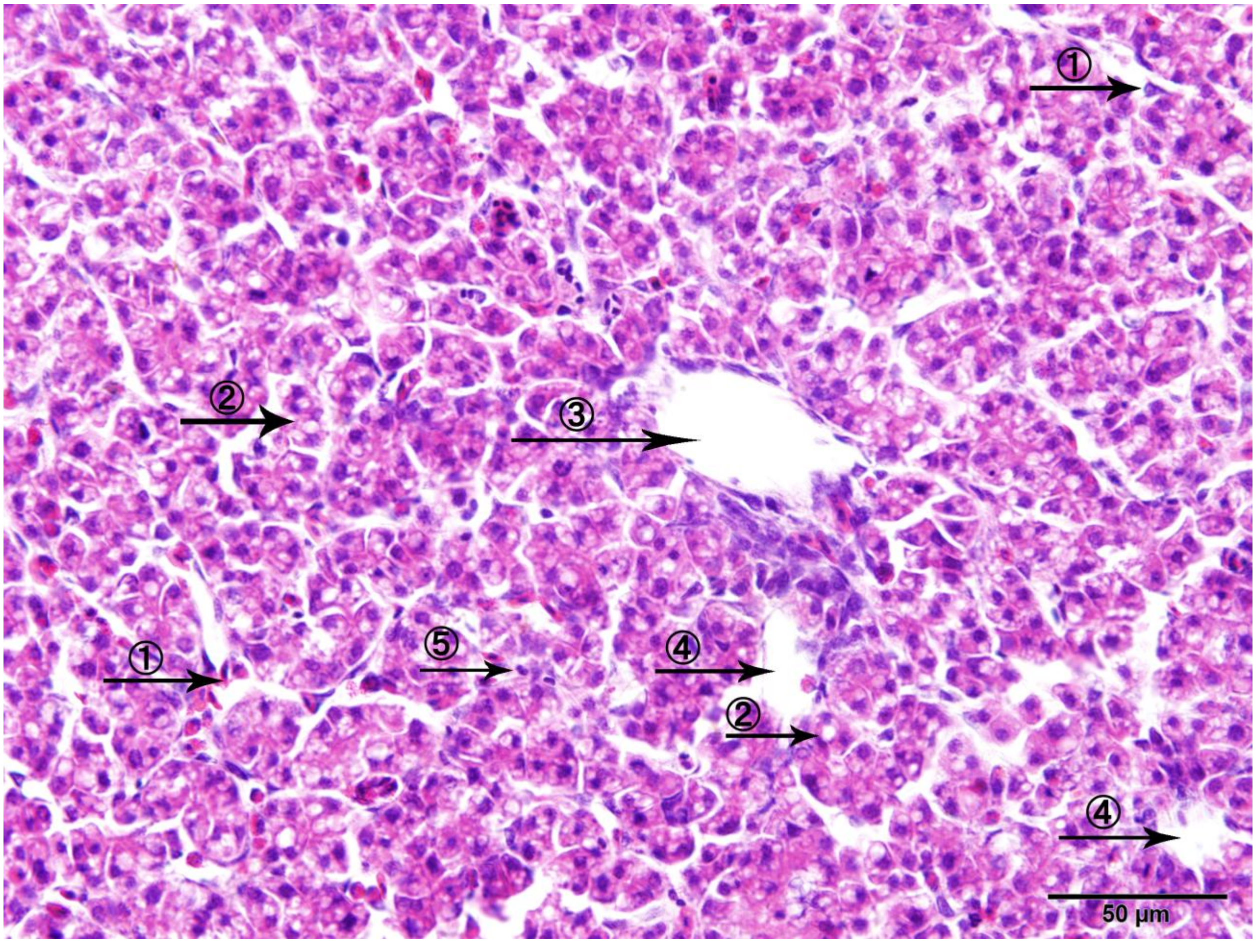

Fig. 1a (at 6 h post LPS stimulation) H&E staining

Enhanced stellate macrophages (Kupffer cells) in perisinusoidal areas ①, diffuse infiltration of fat vacuoles indicating fatty infiltration ②, Central veins ③ and sinusoidal capillaries ④ were both dilated, reduction in size of a few hepatocytes ⑤.

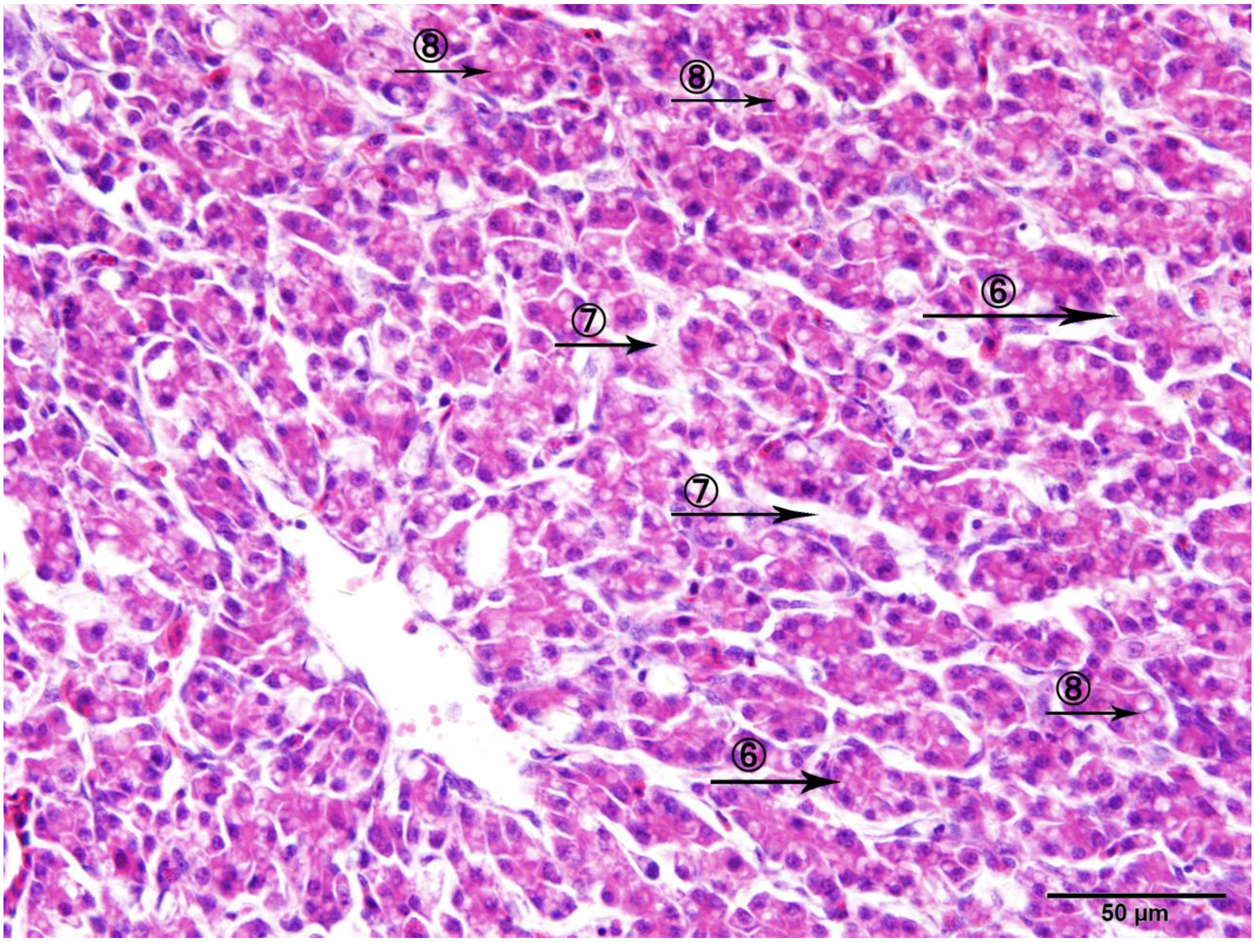

Fig. 1a (at 12 h post LPS stimulation) H&E staining

Liver cells were seen dissociated from each other in hepatic cords ⑥, Heptaic sinusoids were dilated at many places along with fibrocytes proliferation in perisinusoidal areas ⑦, Intracytoplasmic infiltration of variable size and shape fat vacuoles ⑧

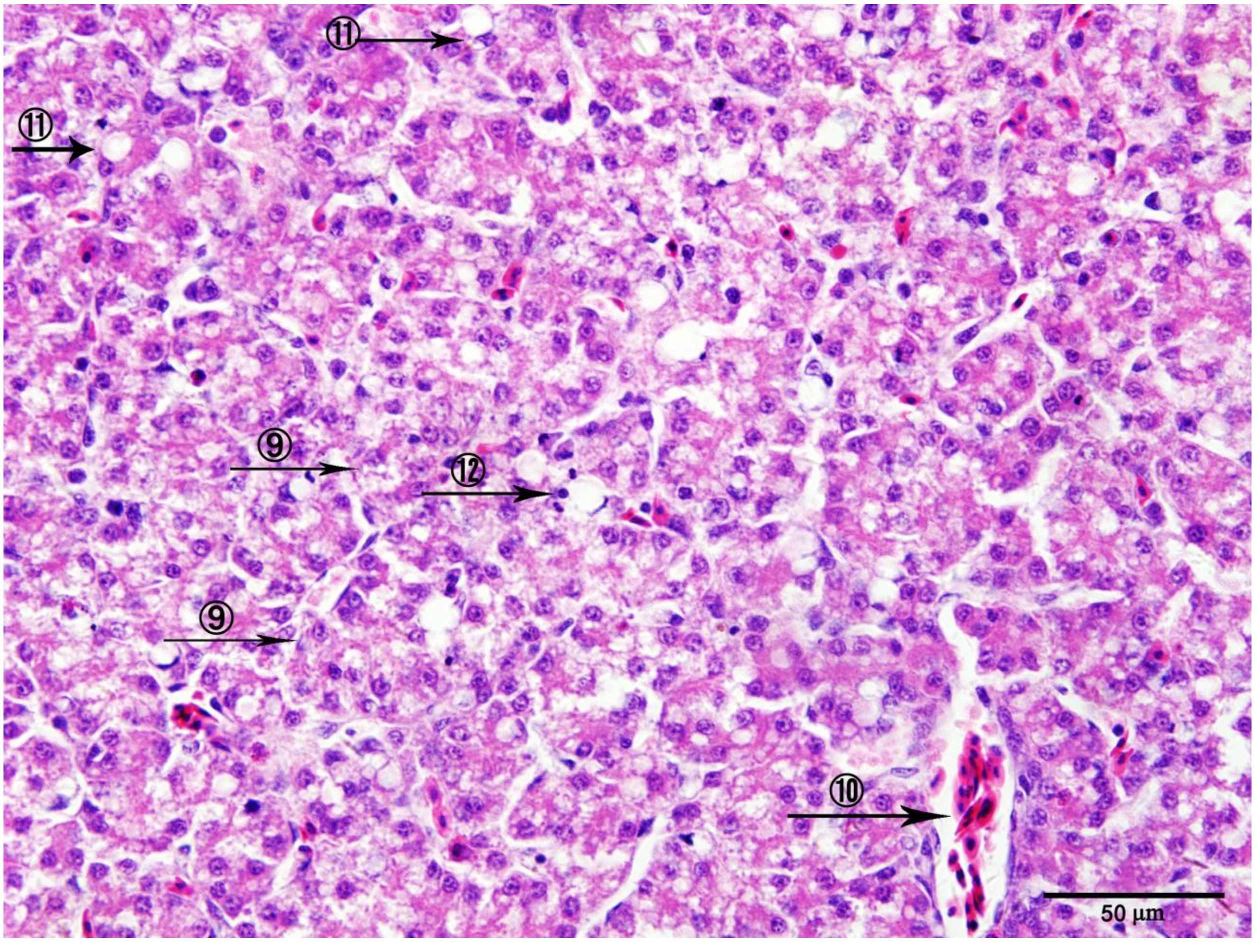

Fig. 1a (at 24 h post LPS stimulation) H&E staining

Hepatic sinusoids were dilated at many places ⑨, infiltration of oval shaped nucleated RBCs showing vascular congestion ⑩ cytoplasmic fat vacuoles have pushed hepatocyte nuclei towards periphery at some places ⑪ and reduction in size of a few hepatocytes ⑫.

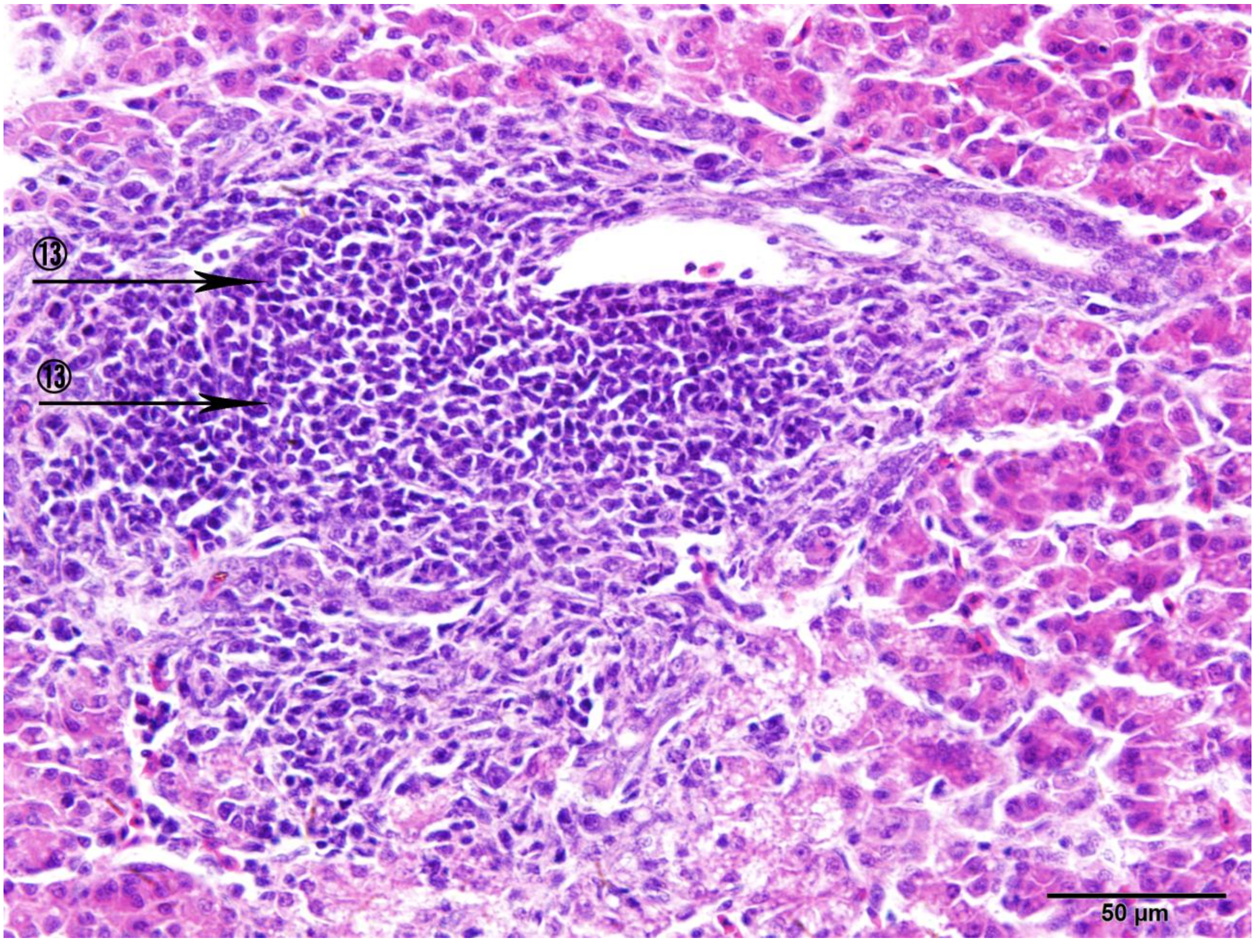

Fig. 1a (at 72 h post LPS stimulation) H&E staining

Obvious pathological changes (inflammatory cells infiltration around the portal area) ⑬
